# The Role of Ultrasound and Computed Tomography in the Evaluation of Subcutaneous Esophageal Bypass in a Dysphagic Patient

**DOI:** 10.1155/2012/827567

**Published:** 2012-12-30

**Authors:** Simone Vetere, Maria Luisa Mennini, Daniele Pironi, Manuela Brighi, Stefano Pontone

**Affiliations:** ^1^Department of Radiological Sciences, Sapienza University of Rome, 00161 Rome, Italy; ^2^Department of Surgical Sciences, Sapienza University of Rome, 00161 Rome, Italy

## Abstract

Several conditions require subcutaneous colon bypass surgery in the esophageal diseases treatment. Esophageal reconstructions are high risk procedures because of their morbidity and mortality rate. Cervical anastomotic strictures, colon transplant redundancy, recurrent dysphagia, intestinal obstruction, regurgitation, and aspiration are the most frequent late complications. The patient assessment should be performed with noninvasive methods in order to prevent long-term complications. We report the use of ultrasound (US) and computed tomography (CT) for evaluating a dysphagic patient, after subcutaneous esophageal bypass. A thorax and upper abdomen contrast media CT study with volume rendering reconstruction was performed in order to evaluate late post operative complications. In addition a US examination, performed after CT scan, was used for the assessment of the colonic wall and its vascularization. The subcutaneous esophageal bypass allowed for an effective ultrasound evaluation with no additional discomfort for the patient. ultrasonography has been shown effective in the esophageal bypass follow up, when subcutaneous colon bypass surgery was performed. The ultrasonography evaluation, also thanks to a Doppler flowmetry, allowed completing the patient assessment without additional invasive procedures or contrast. Thus it may be performed as a first level evaluation or in the follow up of subcutaneous esophageal bypass patients.

## 1. Introduction 

Although gastric reconstruction is the standard procedure [1], esophageal bypass procedures, including substernal gastric bypass surgery and substernal or subcutaneous colon bypass surgery, are performed for multiple benign and malignant esophageal lesions when the first option is not available [2]. In contrast to the other digestive reconstructions, the esophageal surgery may require an extensive mobilization [3, 4]. Thus, the main surgical end-points are to obtain a tension-free cervical anastomosis, using a sufficiently long graft and ensure an optimal blood supply. Esophageal reconstructions are high risk procedures because of their high morbidity and mortality rate. Thus, the benefits of cancer treatment conflict with the poor quality of life. Cervical anastomotic strictures, colon transplant redundancy, recurrent dysphagia, intestinal obstruction, regurgitation, and aspiration, due to loss of the esophageal sphincter, are the most frequent long-term complications [5]. However, the most worrying complication is the graft necrosis due to ischemia [4, 6, 7]. Subcutaneous colon bypass approach is chosen when gastric tubulization method is not accessible. In this case, descending colon and ileocolon [8] are the most common surgical device used [2, 9], even if a visible peristalsis may cause a cosmetic disfigurement and social embarrassment especially in female patients [10]. We report a 53-year-old female case admitted to our department with severe dysphagia as a possible esophageal bypass long-term complication.

## 2. Case Report

**Figure 1:**
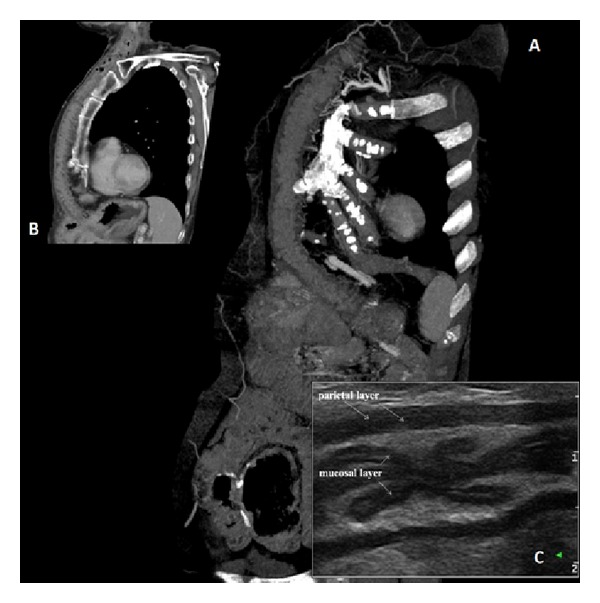
((a)-(b)) Sagittal view CT images show the normal esophageal bypass position without signs of dilatation or ischemic suffering. (c) US image allows an optimal evaluation of mucosal and parietal layers in the whole length of the organ.

**Figure 2:**
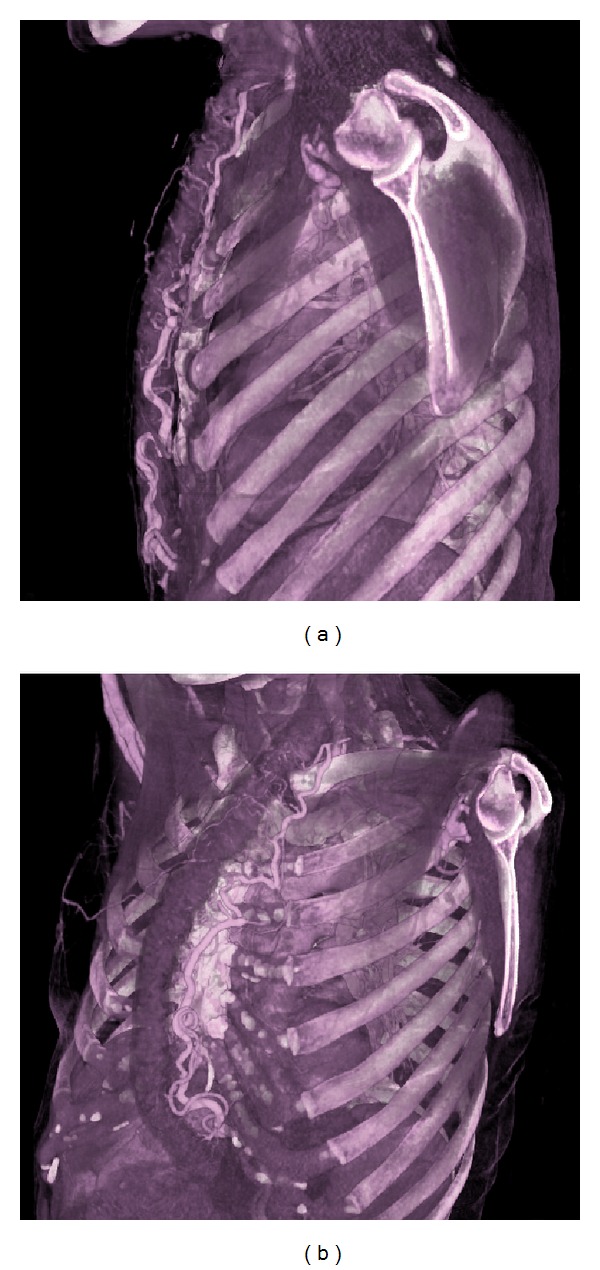
((a)-(b)) CT images with volume rendering reconstruction show the vascular anastomosis condition without stenosis or low-level perfusion signs.

A 53-year-old female was admitted to our department for a severe dysphagia. A subtotal gastrectomy was performed 20 years before for a gastric ulcer. Thus, an oesophageal iatrogenic perforation, during a follow-up endoscopy, was firstly treated with gastrostomy, then, after stabilization, with a subcutaneous oesophageal bypass using left colon. The patient reported dysphagia, resistant to the symptomatic therapy for about 24 hours, without a concomitant trauma or other related events. No specific laboratory test abnormalities or sternal area abnormality were detected during examination. A thorax and upper abdomen contrast media computed tomography (CT) study was performed in order to evaluate late postoperative complications. The exam showed normal position of the esophageal bypass with no signs of dilatation or ischemic suffering (Figures 1(a) and 1(b)) and regular vascular anastomosis condition without stenosis or low-level perfusion signs after volume rendering reconstruction (Figures 2(a) and 2(b)). A 64-section scanner (Sensation Cardiac 64; Siemens, Forchheim, Germany) was performed with the following parameters: section thickness: 0.6 mm; reconstruction interval: 0.5 mm; pitch 0.9; 100 kV; reference tube current: 200 mAs; table feed 40 mm/sec. Considering the contrast enhance method of CT, the patient received iomeron 400 (400 mg of iodine per millilitre, Iomeprol 400; Bracco Imaging, Milan, Italy) injected with an automated dual-rail injector (Stellant; MEDRAD, Warrendale, PA) at a rate of 3 mL/sec. We injected 120 mL of contrast medium and a saline flush of 30 mL injected at the same rate (3 mL/sec). The ultrasound (US) evaluation, performed after CT scan using a Technos MPX, Esaote, Genova, Toshiba Aplio VX, Osaka, Japan, equipped with a high frequency linear probe (7.5–13 MHZ), showed the mucosal and parietal layers of subcutaneous descending colon used in esophageal bypass (Figure 1(c)). The graft condition was confirmed in the US exam without contrast or additional discomfort for the patient. Proton pump inhibitors (PPIs) plus sodium alginate solution have been recommended as symptomatic therapy. This combined assessment, excluding late complications, enabled the rapid patients discharge without hospital admission. During the follow up, four weeks after presentation, the symptoms were greatly reduced together with a well-tolerated therapy.

## 3. Discussion 

The use of gastric tube represent the gold standard for esophageal reconstruction because of their reliable vascularity and the relative technical simplicity. On the other hand, the use of the colon, because of its complexity (three anastomoses) and high risk of necrosis, should be considered as a second choice. In fact, esophageal reconstruction is associated with a high risk of perioperative morbidity and mortality and late complications [4–6, 10, 11]. While the anastomotic leakage is a potentially fatal perioperative complication, the anastomotic strictures, colon transplant redundancy, severe dysphagia, intestinal obstruction, regurgitation, and aspiration are the most frequent long-term complications [5]. The relevance of proper blood circulation is also demonstrated by several studies [7, 10, 12] that assessed the complications rate with regard to the vascularization of the colonic graft, especially in the use of right colon. Thus, whatever the potential complication, the arterial blood supply to the graft must be assessed in patients presenting worrisome clinical signs. The use of CT scan, endoscopy, and interventional radiology, promoting the early diagnosis and treatment of complications, has significantly reduced the mortality rate associated with this surgery. Unfortunately, although no information is reported on the graft blood supply, the water-soluble contrast studies remain the most common, routine diagnostic test during esophageal reconstruction follow up. Recently advances in CT technology provided high resolution and more accurate images, such as multiplanar reconstruction, three-dimensional reconstruction, maximum intensity projection (MIP), and virtual endoscopy. In this way, advantages over conventional CT images in diagnosing were obtained. Particularly, by volume-rendering technique reconstructing and MIP, a highly defined and detailed overview of the blood supply can be visualized. In our case, due to the substernal location of colonic graft, we decided to perform an US assessment. The US evaluation, also thanks to a Doppler flowmetry, allowed completing the patient assessment without additional invasive procedures or contrast. In fact, US is a noninvasive technique for the evaluation of organ wall, allowing an accurate assessment of the colonic wall vascularization. Thus, it may be performed either as a first level evaluation or in the follow up of subcutaneous esophageal bypass patients, complementary to CT.
